# What Can the Organization of the Brain’s Default Mode Network Tell us About Self-Knowledge?

**DOI:** 10.3389/fnhum.2013.00391

**Published:** 2013-07-17

**Authors:** Joseph M. Moran, William M. Kelley, Todd F. Heatherton

**Affiliations:** ^1^U.S. Army Natick Soldier Research, Development, and Engineering Center, Natick, MA, USA; ^2^Center for Brain Science, Harvard University, Cambridge, MA, USA; ^3^Psychological and Brain Sciences, Dartmouth College, Hanover, NH, USA

**Keywords:** cognitive neuroscience, self-reflection, medial prefrontal cortex, posterior cingulate cortex, default mode network

## Abstract

Understanding ourselves has been a fundamental topic for psychologists and philosophers alike. In this paper we review the evidence linking specific brain structures to self-reflection. The brain regions most associated with self-reflection are the posterior cingulate and medial prefrontal (mPFC) cortices, together known as the cortical midline structures (CMSs). We review evidence arguing that self-reflection is special in memory, while noting that these brain regions are often engaged when we think about others in our social worlds. Based on the CMSs’ patterns of connectivity and activity, we speculate about three possible interpretations of their role in supporting self-reflection that are somewhat overlapping, and not intended to be mutually exclusive. First, self may be a powerful, but ordinary case for a cognitive system specialized for thinking about people. Second, mPFC may serve as a processing “hub,” binding together information from all sensory modalities with internally generated information. Third, mPFC may serve as a cortical director of thought, helping to guide moment-by-moment conscious processing. Suggestions are made for future research avenues aimed at testing such possibilities.

How do we know what we are like? How do we determine the boundaries between ourselves and the world around us? How do we know what is ours, and what is not? Questions like these have engaged philosophers for millennia, and psychological scientists throughout psychology’s relatively brief history. Great progress has been made by practitioners of these fields, who have recently been joined by neuroscientists bearing the promise of going beyond introspection, self-report, and behavior to the source of our sense of self, the brain. This work is theoretically useful in at least two ways. First, it enables characterization of how the brain implements the psychological process(es) of self-reflection, allowing for links between the neural and psychological levels of analysis. Second, it may suggest new ways to interpret and modify accounts of self-reflection at the psychological-level, allowing neural-level data to influence psychological-level theorizing. Wielding two major empirical breakthroughs, cognitive neuroscientists have made significant headway in understanding how the brain gives rise to a sense of self, revealing surprising knowledge about the organization of the neuronal networks responsible for self-reflection.

## The Default Mode Network and Self-Reflection

In brief, these breakthroughs consisted first of the discovery of what has come to be known as the default mode network (Shulman et al., 1997; Raichle et al., 2001), and second of the independent identification that a subset of these brain regions are enlisted when we engage in self-reflection (Gusnard et al., 2001; Johnson et al., 2002; Kelley et al., 2002). To be clear, this network’s involvement is observed most closely during the psychological task of reflecting on one’s personalities and characteristics (self-reflection), rather than during self-recognition, thinking of the self-concept, or thinking about self-esteem, for example. As such, this paper will focus on self at the level of self-reflection and the neural networks responsible for this task. The set of regions contributing to self-reflection consists primarily of the medial prefrontal cortex (mPFC), encompassing the medial surface of the medial frontal gyrus [Brodmann’s Areas (BAs) 8 and 10], and the medial parietal cortex, roughly encompassing the retrosplenial and posterior aspects of the cingulate cortex, the area bounded at the anterior by the paracentral lobule, and at the posterior by the parieto-occipital sulcus (BAs 23, 31, 7). For ease of reference, we will refer to this medial parietal cluster together as posterior cingulate cortex (pCC). These regions have come to be known together as the cortical midline structures (CMSs) (Northoff and Bermpohl, 2004), and are the regions most closely associated with self-reflection in meta-analyses (e.g., Northoff et al., 2006; Qin and Northoff, 2011).

The default mode network concept arose to explain the puzzling observation that when subjects rest quietly with eyes closed, CMS activity is elevated (as measured by positron emission tomography), along with that of anterior temporal lobes and lateral parietal cortices (Shulman et al., 1997). This set of regions is more active when people rest than when they are engaged in goal-directed tasks, and display *functional connectivity*: these regions’ activity rises and falls together during the normal course of cognitive engagement and disengagement from the external world (Greicius et al., 2003; Fox et al., 2005). This led Raichle and colleagues to propose that this set of regions formed a default mode network; a network that may serve to generate internal mental stimuli and pay attention to our stream of consciousness, but whose activity is attenuated when we turn our attention to the outside world (as in goal-directed tasks) (Gusnard et al., 2001; Raichle et al., 2001). Following these observations, several labs demonstrated direct overlap between the brain regions engaged during rest and during self-reflection (Wicker et al., 2003; D’Argembeau et al., 2005; Schneider et al., 2008; Whitfield-Gabrieli et al., 2011). This relation is further supported by a meta-analysis (Qin and Northoff, 2011), which reported that the same finding occurred across many studies.

## Medial Prefrontal Cortex

Several aspects of these regions’ neuroanatomy may support these well-characterized roles. mPFC is larger than any other prefrontal region in humans (Ongur et al., 2003). By proportion, it covers more of the cortex in humans and has more space available for connections with other supramodal areas than in other primates (Semendeferi et al., 2001). It has a greater density of dendritic spines (69% more on average than primary sensory cortex) and smaller density of cell bodies on the average than other cortical regions, suggesting more complex associative processing (Jacobs et al., 2001). Finally, mPFC is almost exclusively interconnected with other heteromodal processing regions in the prefrontal cortex (Barbas and Pandya, 1989; Petrides and Pandya, 1999), anterior temporal cortex (Amaral and Price, 1984; Morán et al., 1987), and the cingulate gyrus (Morecraft and Van Hoesen, 1993; Arikuni et al., 1994). Most of these connections are reciprocal in nature (Passingham et al., 2002).

These regions are considered to be part of the “social brain”: a network implicated by neuroimaging and lesion work in representing the people that populate our social worlds (Adolphs, 2001; Heatherton, 2011; Lewis et al., 2011). mPFC’s enlargement in humans, preponderance of interconnections rather than cell bodies, and connections with other “social brain” nodes are all features that point toward a role in social abstraction, a skill for which humans are evidently selected (Dunbar, 2009). Indeed, humans form much larger social networks than do other animals (Dunbar, 1998). Lewis et al. (2011) showed further that the size of particular mPFC regions is correlated both with the degree to which we are able to represent multiple others’ viewpoints and the size of our social networks. Underscoring the role of mPFC in social processing in general, and self-processing in specific, a recent meta-analysis further subdivides mPFC into ventral and dorsal aspects (Denny et al., 2012; Wagner et al., 2012), showing that ventral mPFC responds more to self, and dorsal mPFC responds more to others.

## Medial Parietal Cortex

Posterior cingulate cortex shares many reciprocal connections with mPFC. In addition, the subregions of pCC are reciprocally connected with one another in a bilateral manner (Cavanna and Trimble, 2006). Along with mPFC, pCC is disproportionately large in humans relative to non-human primates (Goldman-Rakic, 1987). pCC shares many connections with subcortical and cortical regions and serves as “association cortex,” allowing the brain to “integrate both external and self-generated information and to produce much of the mental activity that characterizes *Homo sapiens*” (Cavanna and Trimble, 2006, p. 568). This set of neuroanatomical features suggests that these regions would be good candidates for those able to perform the inward-focusing and self-generation of stimuli that constitute mental activity when we are not focused on the external world (Mason et al., 2007; Smallwood et al., 2008). That these regions are disproportionately developed in humans, and that humans congregate in the largest social networks, suggests that much of this mental activity at rest might be about ourselves and others.

If we were to plan to design a system that would be able to retain information about itself, to determine what is and is not self, and to update that store of information in a flexible and goal-dependent manner, we could do worse than to outfit it with the array of connections and features that are possessed by the CMS. While the neuroanatomical evidence is certainly suggestive of a set of regions that are specialized for self-reflection, stronger evidence has emerged in cognitive neuroscience. Work that we and others have done has repeatedly demonstrated that reflecting on the self engages the CMS relative to reflecting about (certain) other people, or non-social classes of stimuli (Craik et al., 1999; Johnson et al., 2002; Kelley et al., 2002; Heatherton et al., 2006; Moran et al., 2011; Whitfield-Gabrieli et al., 2011). This work has supported the idea that the self is a special cognitive structure, providing a superordinate means by which information can be encoded into memory (Fossati et al., 2004; Macrae et al., 2004). This position is further supported by neuropsychological work from Klein et al. (1999) that revealed a post-lesion dissociation in patients’ abilities to form memories about the self versus about general semantic categories. The theoretical position that self-is-special is in direct contrast to the notion that the self is a “powerful, but ordinary” structure in memory; a view which suggests that our improved memory for information encoded in reference to the self is simply a result of the greater familiarity of the self-concept (Greenwald and Banaji, 1989), but that the semantic structures of self are no different from the semantic structures of sailboats and silver jewelry. Even though the cognitive neuroscience evidence strongly supports the self-is-special view, Denny et al.’s (2012) meta-analytical finding of a dorsal-ventral axis along which mPFC appears to be differentiated for other- and self-representation appears contradictory. Why is it the case that, on the one hand, our neural representations of self and other are so closely allied, but on the other hand these representations occur in regions of the cortex distinct from (and largely anatomically disconnected from) those networks that are engaged when we reflect about non-social sources of information?

## Why Does Self-Reflection Engage the Cortical Midline Structures?

**Figure 1:**
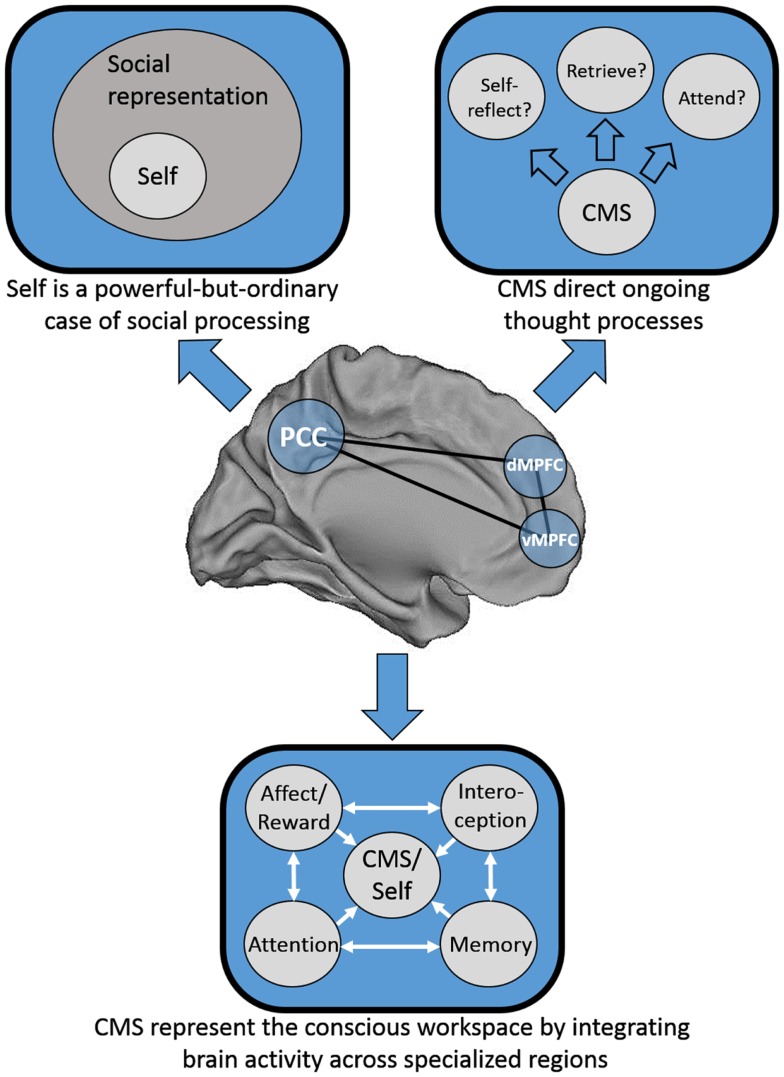
**Schematic representation of three possible distinct, but not mutually exclusive models of cortical midline structure (CMS) function**. Top left: the CMS are specialized for representing social information, of which the self is a powerful-but-ordinary subset. Top right: the CMS serve as a set of regions responsible for the direction of our thought processes on a moment-to-moment basis. Bottom: the CMS serve as a hub integrating information from disparate neural processing systems into a “conscious workspace.”

We consider three possible explanations for this pattern of results that are speculative, not intended to be mutually exclusive, and are at least partially overlapping. First, one possibility is that Greenwald and Banaji (1989) may have been half-right: it may be that social information is special, and that the self is a powerful-but-ordinary *social* knowledge structure. Second, Heatherton (2011) has proposed that mPFC serves as a “hub,” binding together heavily processed information from secondary sensory areas from each of the senses with internally generated information to represent the conscious “workspace.” Third, mPFC may act in a meta-cognitive fashion by guiding our moment-to-moment thought processes; in essence, in deciding what to think about next. See Figure 1 for a schematic representation of each of these models.

## Is the Self a Powerful-But-Ordinary Social Construct?

On the self is a powerful-but-ordinary social construct view, the CMS could be seen as representing social information *per se*, and their seeming selectivity for self-relevant information might simply represent an extreme case of social information processing about a social target (the self) that by definition is more *familiar* than all other social targets. The overarching view of simulation theory (Gordon, 1986) is that in order to understand others we run a mental simulation of how we might act in given social situations. Conversely, the emerging discipline of neural hermeneutics (Gallotti and Frith, 2013) suggests that in order to understand ourselves, we pay close attention to the social behavior of others. Both of these viewpoints converge on the idea that the self might be a powerful, but ordinary social target.

One obvious prediction of this idea is that the CMS might be differentially engaged by the representation of (and processing about) social targets that are differentially familiar to us. Familiarity contains the concepts of both closeness and similarity: close individuals are those we feel close to (including family and friends), whereas similar individuals are those who share characteristics with us (like members of our race, political affiliation, or age group). Indeed, in Qin and Northoff’s (2011) meta-analysis, they observe that stimulus *familiarity* drives activation in a similar ventral mPFC region just as much as does self-reflection. In addition, Denny et al.’s (2012) meta-analysis shows that ventral aspects of mPFC are preferentially engaged by reflecting on the self versus others. If this region is sensitive to the familiarity (or “selfness”) of social information, then it should respond more to information that is more self-relevant than not. Several studies have found such a pattern of results (e.g., Phan et al., 2004; Moran et al., 2006). Indeed, Mitchell et al. (2006) observed that social targets manipulated to be similar to the self engaged this ventral mPFC region, whereas social targets manipulated to be dissimilar to the self engaged dorsal mPFC. Krienen et al. (2010) clarified Mitchell et al.’s findings by demonstrating that the driver of activation in mPFC was closeness rather than similarity *per se*, suggesting that the familiarity of repeated exposure to individuals drives their self-relevance.

Converging on this idea, a series of studies investigating self-reflection in different cultures have provided support for the notion that in individuals whose cultures are more interdependent, the same ventral mPFC region does not differentiate thinking about self from thinking about close family members (like participants’ own mothers) (Zhang et al., 2006; Zhu et al., 2007; Chen et al., 2013), but that this does not necessarily hold true in Western, more independent cultures (Kelley et al., 2002; Kjaer et al., 2002; Heatherton et al., 2006; Vanderwal et al., 2008). These cross-cultural findings are best interpreted in the context of recent criticisms suggesting that standard delineations between Western and Eastern cultures are not as clear-cut as has been suggested (Martinez Mateo et al., 2013). In this context, Moran et al. (2011) provide data that clarify the distinction between independent and interdependent cultures. In their paper, consideration of one’s mother’s personality traits, but not her physical characteristics produced activation levels midway between those of thinking about one’s own traits versus those of former US President, George W. Bush. To the degree that we represent the traits of a close other as being like our own (rather than their physical characteristics), this suggests again that “selfness” may be driving this difference in ventral mPFC). Considered as a unit, these lines of research reveal a quantitative dimension along which social targets of greater familiarity activate ventral mPFC to a greater degree, with the self sitting at the top as the most familiar social target of all.

A further prediction of the notion that the CMS are specialized for *social* processing (and that the self is a powerful subset of such processing) is that we might be able to differentiate their relative contributions along lines in which thinking about ourselves and thinking about others naturally cleave. To the degree that our representations of ourselves are first-person, and our representations of others are third-person, one would imagine that neural systems implicated in social processing that preferentially receive *visual* information would be more responsive to third-person representations. Based on the patterns of connectivity that we introduced at the beginning of this paper, it should be clear that the regions of pCC implicated in the default mode (and in self-reflection) are strongly linked to regions that create complex visual representations. Indeed, Raichle et al. (2001) advocate for a domain-general role for the pCC regions in providing complex visual representations to consciousness. Other work in cognitive neuroscience supports and extends this view, showing via meta-analysis that pCC regions participate in a network engaged in autobiographical memory, prospective future thinking, and navigation (Spreng et al., 2009). All such tasks require complex visual representation, and it is interesting that mPFC did not emerge in this meta-analysis. More direct evidence in support of the idea that pCC supports the third- rather than first-person representations more common in thinking about others rather than the self comes again from the meta-analysis of Denny et al. (2012). In their paper, they found across 107 studies that the precuneus was more active when participants thought about others than when they thought about themselves. Single-study evidence of the idea that visual rather than conceptual representations of people engage pCC comes from Moran et al. (2011), who showed that thinking about social targets’ appearance (e.g., Does George W. Bush have a beard?) versus thinking about their character traits (e.g., Is George W. Bush kind?) produces more activation in pCC. This relationship also held true when the social target was the self. Direct investigations of adopting third- versus first-person perspectives have also shown greater pCC involvement during third-person perspective taking (Ruby and Decety, 2001).

## Is Medial Prefrontal Cortex a Hub for Integrating Internal and External Information?

Our second possibility is that the ventral mPFC region identified by Heatherton (2011) serves as a hub that integrates internal and external information into a conscious workspace. On this view, self-reflection would be the canonical task for such a region because it so strongly requires the flexible and ongoing integration of our own knowledge about ourselves with our ever-changing knowledge gained from our sense organs about how we are interacting with the environment, and about how social actors in our environment think about us. Thinking about those social actors independent from ourselves (theory of mind) would drive this machinery to a lesser degree (but still more than thinking about non-social aspects of the world) because rapid and complex integration of sensory, external, and non-sensory conceptual knowledge is required to understand others’ goals, intentions, and beliefs, whereas such dynamic processing is much less necessary for thinking about tools or cars or jewelry. This sort of integration into a conscious workspace is also a hallmark of the cognitive processes engaged during “rest,” and engendered by the default mode of brain functioning.

## Is Medial Prefrontal Cortex Specialized for Directing Conscious Thought Processes?

Finally, our third possibility is that the ventral mPFC region identified in self-reflection tasks is specialized for helping to decide in which direction our thought processes should proceed. The convergence of heavily processed external sensory inputs with internally generated inputs would also support this view, which of course is not mutually exclusive with the view that mPFC serves as a hub for integration of information from disparate neural processing units. To the degree that deciding where our thoughts should go is a representational process, and that reflection on those thoughts (and our enduring personality traits) is a meta-representational version of the same process, one would imagine that a system with such functional-anatomic properties would be well-placed to perform both conscious direction of thoughts and self-reflection. That rest and self-reflection so consistently overlap (Qin and Northoff, 2011; Whitfield-Gabrieli et al., 2011) suggests that being free to direct our own thoughts (i.e., not responding directly to the environment or an experimenter-provided task) is a state that mimics the natural process observed when we are asked to reflect directly on our own selves. A prediction of this viewpoint is that decision-making might be tied to activity in the CMS, and indeed research shows that CMS activity predicts freely made decisions up to 7 s before participants indicate becoming aware of the decision having been made (Soon et al., 2008). This third possibility thus may account for the still-puzzling observation that the mPFC is perhaps the most important actor in the brain’s default mode network, which itself perhaps serves as a proxy for our ongoing conscious awareness of both our internal and external words. This conjecture awaits empirical investigation however, not least because sampling the ongoing representational processes of the default mode requires disrupting such processes.

## Conclusion

In summary, we have speculated about several different explanations for the observation that the CMS are observed so consistently to participate in self-reflection. Neuroanatomical connectivity suggests that these regions are heteromodal association areas that derive much of their inputs from upstream regions associated with social information processing, and that pCC in particular gains its inputs from regions of the brain responsible for complex visual representations. Because these regions are associated with social processing, are developed strongly in humans relative to other animals, and humans travel in much larger social networks than do other animals, we speculate that they may form the basis of a special neurocognitive system evolved for social processing. More fundamental characterizations of this system suggest that the anterior midline structure, mPFC, is in fact a domain-general region dedicated as a hub of information processing about the internal and external worlds, and relatedly, that the purpose of such a confluence of representations is to direct our conscious awareness from one moment to the next, switching flexibly between representations of our internal mental life and of the world around us. On this view, mPFC’s seeming specialization for social information processing merely reflects its response to stimuli (self and others) that drive the integration of internal and external information sources more strongly than non-social stimuli.

Much research remains to be done to gain greater understanding of how and why the self, other social targets, and the default mode of thought are related to one another, and why they so reliably involve the CMS. Initial support for the idea that mPFC regions might be *necessary* for self-reflection comes from a study with patients with ventral mPFC damage at the site implicated by Kelley et al. (2002) as being maximally involved in self-reflection (Philippi et al., 2012). These patients did not show the self-reference effect in memory, suggesting that mPFC is necessary for encoding information in relation to oneself. Emerging advances in TMS may allow researchers to target more closely these regions for temporary, reversible lesions, or for theta-burst stimulation for temporary increases in excitability of these regions (Vernet et al., 2013). Such studies could provide more controlled evidence to determine whether these regions are *necessary* for reflection about self and other. In parallel, advances in real-time fMRI techniques (deCharms et al., 2004; Hinds et al., 2011) allow for the exquisite control of presentation parameters, such that we can manipulate when participants are asked to reflect on self and others to moments when activation in either mPFC or pCC are high or low, and determine with a great degree of accuracy what effects natural fluctuations in the default mode at any given moment might have on our abilities to accurately represent ourselves.

## Conflict of Interest Statement

The authors declare that the research was conducted in the absence of any commercial or financial relationships that could be construed as a potential conflict of interest.
